# Congenital bronchial atresia complicated by a lung abscess due to *Aspergillus fumigatus*: a case report

**DOI:** 10.1186/s12890-020-01367-5

**Published:** 2021-01-06

**Authors:** Gaku Kuwabara, Risa Sone, Waki Imoto, Kazushi Yamairi, Wataru Shibata, Kazuhiro Oshima, Koichi Yamada, Kazuhiro Yamada, Tetsuya Watanabe, Kazuhisa Asai, Hiroaki Komatsu, Nobuhiro Izumi, Noritoshi Nishiyama, Tomoya Kawaguchi, Hiroshi Kakeya

**Affiliations:** 1 Department of Infection Control Science, Graduate School of Medicine, Osaka City University, 1-4-3, Asahi-machi, Abeno-ku, Osaka-shi, Osaka, 545-8585 Japan; 2 Department of Respiratory Medicine, Graduate School of Medicine, Osaka City University, 1-4-3, Asahi-machi, Abeno-ku, Osaka-shi, Osaka, 545-8585 Japan; 3 Department of General Thoracic Surgery, Graduate School of Medicine, Osaka City University, 1-4-3, Asahi-machi, Abeno-ku, Osaka-shi, Osaka, 545-8585 Japan

**Keywords:** Congenital bronchial atresia, Lung abscess, Fungal infection, *Aspergillus fumigatus*

## Abstract

**Background:**

Congenital bronchial atresia is a rare pulmonary abnormality characterized by the disrupted communication between the central and the peripheral bronchus and is typically asymptomatic. Although it can be symptomatic especially when infections occur in the involved areas, fungal infections are rare complications in patients with bronchial atresia. We report a case of congenital bronchial atresia complicated by a fungal infection.

**Case presentation:**

A 30-year-old man with no previous history of immune dysfunction was brought to a nearby hospital and diagnosed with a left lung abscess. Although antimicrobial treatment was administered, it was ineffective, and he was transferred to our hospital. Since diagnostic imaging findings and bronchoscopy suggested congenital bronchial atresia and a fungal infection, he was treated with voriconazole and surgical resection was subsequently performed. A tissue culture detected *Aspergillus fumigatus* and histopathological findings were compatible with bronchial atresia. After discharge, he remained well and voriconazole was discontinued 5 months after the initiation of therapy.

**Conclusion:**

Bronchial atresia is a rare disease that is seldom complicated by a fungal infection, which is also a rare complication; however, physicians should consider fungal infections in patients with bronchial atresia who present with infections resistant to antimicrobial treatment.

## Background

Congenital bronchial atresia is a rare pulmonary abnormality typically characterized by segmental or lobar emphysema with or without mucoid impaction from the obliteration of the bronchus [1]. Bronchial atresia is usually asymptomatic and is coincidentally found in most cases. It can become symptomatic when infections occur; however, they are thought to rarely occur in patients with bronchial atresia. Here, we present a unique case of congenital bronchial atresia complicated by lung abscess due to *Aspergillus fumigatus*.

## Case presentation

A 30-year-old male with no medical history was admitted to the nearby hospital with a worsening cough and low-grade fever that had persisted for a month. Chest computed tomography (CT) revealed an abscess in the left lower lobe of his lung, and clinical laboratory tests showed elevated white blood cells of 13,200/μl (normal range, 4000–8000/μl) and C-reactive protein of 5.97 mg/dl (normal range, < 0.40 mg/dl). Treatment with sulbactam/ampicillin was initiated. Since no improvements were confirmed by the imaging and laboratory findings, antimicrobial treatment was changed to tazobactam/piperacillin on day 3 post admission, and was subsequently changed to meropenem (MEPM) on day 25 post admission. Treatment with voriconazole (VRCZ) was added on day 17 post admission because his β-D-glucan level reached to 123 pg/ml (normal range, < 20 pg/ml). However, it was discontinued 8 days later. Transbronchial biopsy and bronchoalveolar lavage performed to assess the mass did not detect malignancy or significant levels of bacteria, including mycobacteria and fungi. On day 27, the patient was referred to our tertiary center for further examination and treatment by specialists in respirology and infection and potential thoracic surgery. Since the definitive diagnosis could not be made, the patient was transferred to our hospital for further examination and treatment on day 27.

**Fig. 1:**
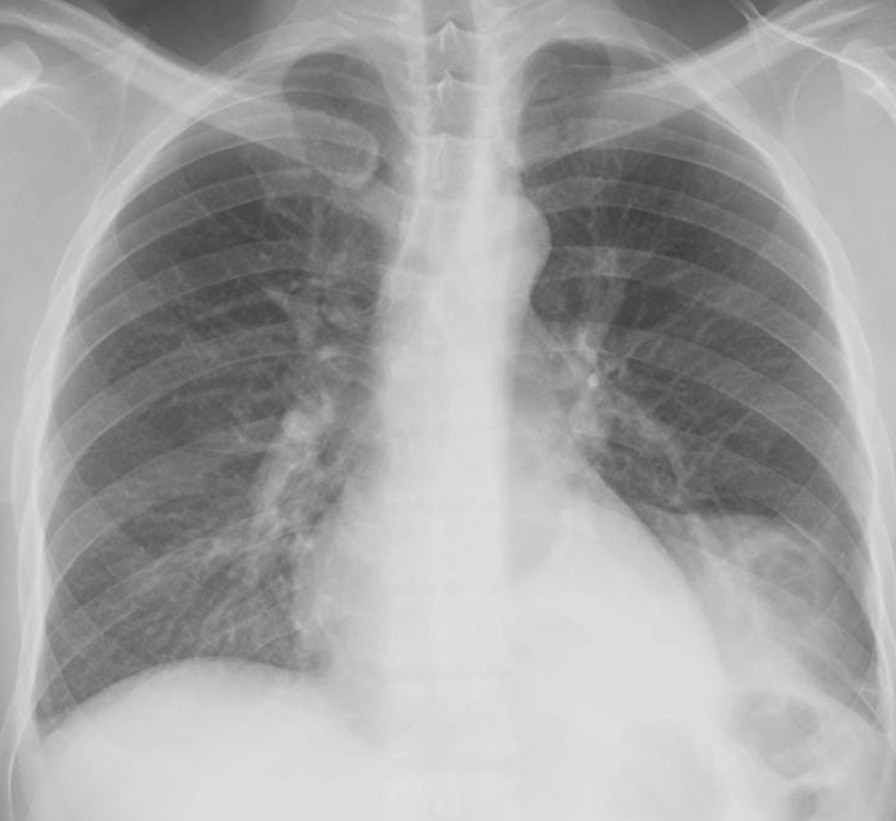
Chest X-ray performed on admission showing an infiltrative shadow in the left lower lung field

**Fig. 2:**
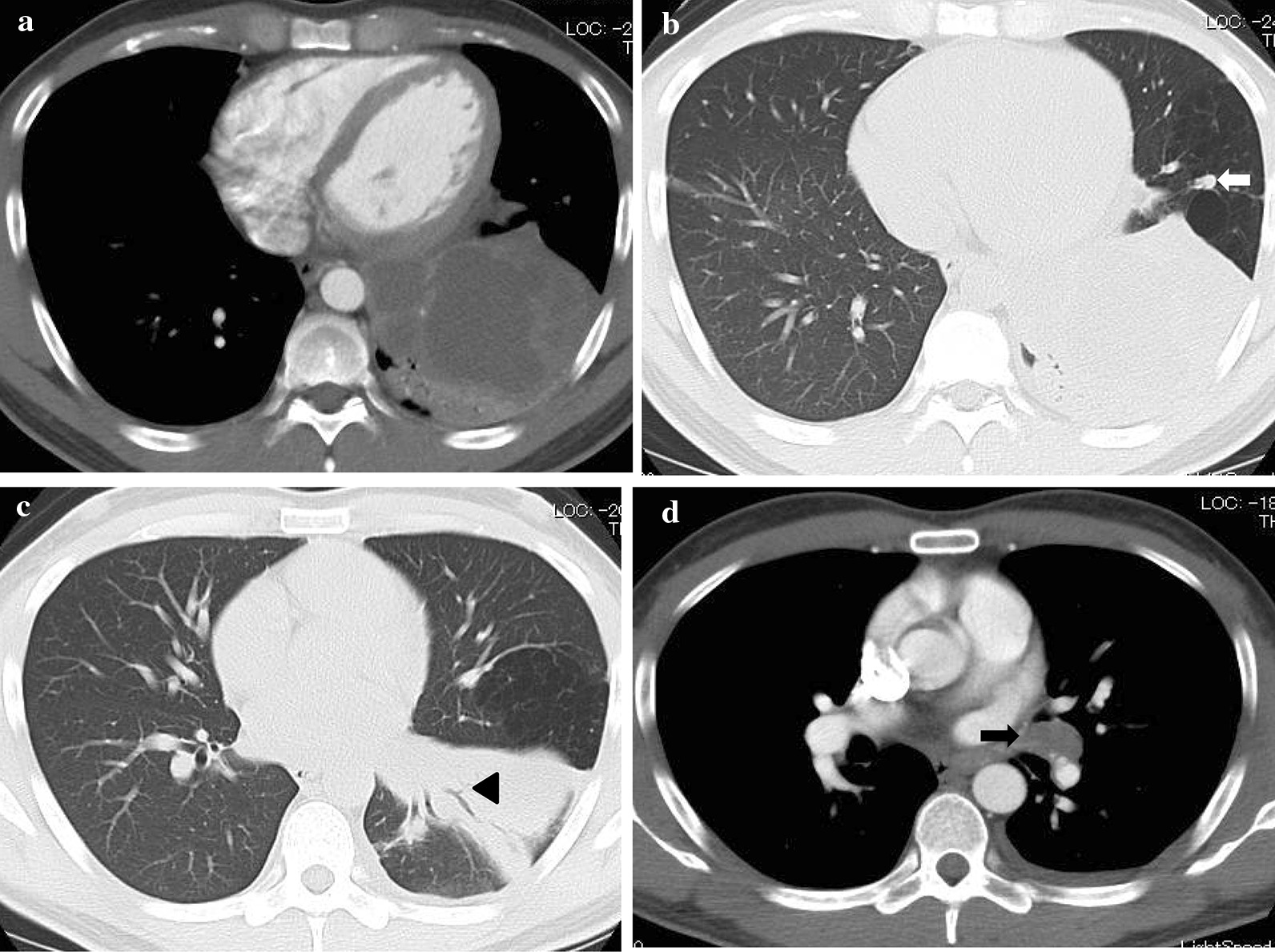
Contrast-enhanced CT performed on admission revealing an abscess, emphysematous changes, ectatic bronchi, a nodular lesion that was considered a mucoid impaction of the lingular segment (arrow), and disrupted bronchus within the left lower lobe (arrowhead) (**a**–**c**). A small mass lesion near the left second carina (arrow) is also shown (**d**)

**Fig. 3:**
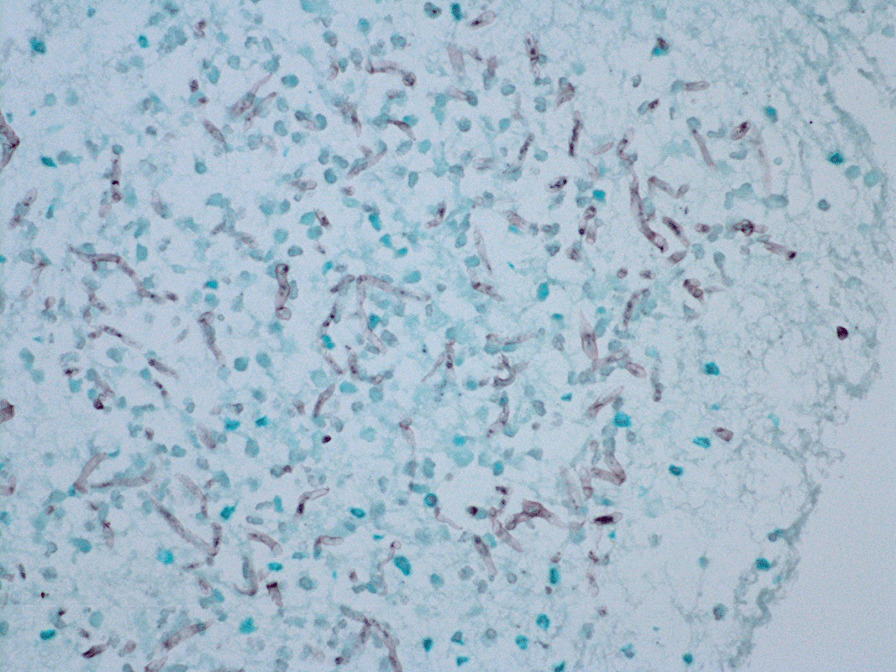
Histopathological assessment of the mass lesion near the left second carina obtained via endobronchial ultrasound-guided transbronchial needle aspiration revealed the presence of filamentous fungi (Grocott staining, × 40)

**Fig. 4:**
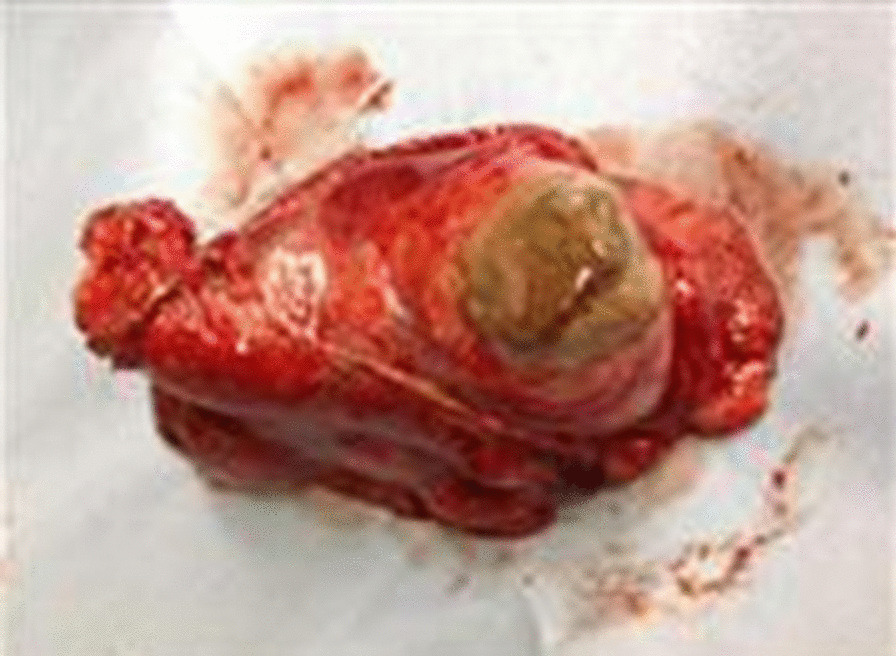
The lingular and basal segments of the left lung were resected, and abscess was macroscopically confirmed

**Fig. 5:**
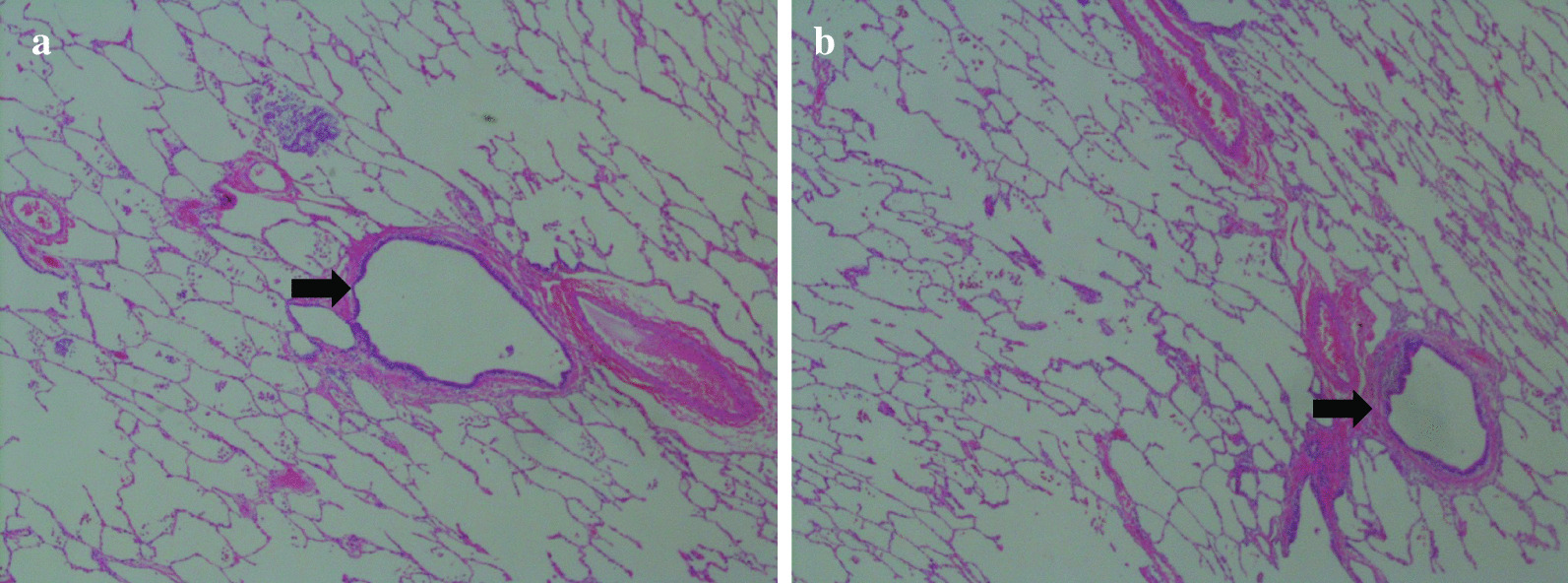
Histopathological assessment of the resected lung showed emphysematous change and ectatic bronchus (arrows) of left basal segments (**a**, **b**) (Hematoxylin and Eosin staining, × 40)

On admission, his vital signs were as follows: a body temperature of 36.6 °C, blood pressure of 120/66 mmHg, heart rate of 84 beats per minute, and a respiratory rate of 12 breaths per minute. The arterial oxygen saturation measured via pulse oximetry was 98% in the examination room. A physical examination including respiratory sound revealed no significant finding. Clinical laboratory tests showed that the patient had elevated C-reactive protein levels of 15.56 mg/dl (normal range, < 0.40 mg/dl) and his *Aspergillus* antigen test was positive (cut-off index of 3.5). Human immunodeficiency virus antibody and interferon-gamma releasing assay were negative. In addition, the β-D-glucan level decreased to a normal range (14.7 pg/ml). Chest X-ray and contrast-enhanced CT were performed on admission. Chest X-ray showed an infiltrative shadow in the left lower lung field (Fig. 1), and CT showed a massive lesion in the left lower lobe of the lung, emphysematous changes, ectatic bronchi, and a nodular lesion that was thought to contain a mucoid impaction within its lingular segment. The bronchus was disrupted proximal to the mass lesion of the left lower lobe (Fig. 2a–c). Consequently, bronchial atresia in the inferior and anterior basal segments of the left lung was suspected based on imaging findings. Since the presence of a malignant lesion could not be excluded, bronchoscopy was performed on day 2 after transfer to our hospital. An endobronchial ultrasound-guided transbronchial needle aspiration was performed on the small mass lesion near the left second carina (Fig. 2d). The disrupted bronchus could not be confirmed via bronchoscopy because the disrupted site was located too peripherally. Histopathological analyses revealed no malignant findings, but presence of a filamentous fungus was indicated in Grocott staining (Fig. 3). Although cultures did not detect any microorganisms, based on these findings, a lung abscess due to *Aspergillus* spp. was suspected. Therefore, VRCZ was reintroduced on day 9 after transfer. To perform treatment and confirm the suspected diagnosis of a lung abscess due to an *Aspergillus* infection, the patient underwent surgical resection of the lingular and basal segments of the left lung on day 22 after transfer. The abscess formation was macroscopically confirmed (Fig. 4), and histopathological examination revealed emphysematous changes and ectatic bronchi within the lingular and left basal segments (Fig. 5a, b). These findings were compatible with bronchial atresia [2], and no malignant lesions were confirmed. Although histopathological analyses did not reveal filamentous fungus, a tissue culture resulted in *A. fumigatus* growth. MEPM and intravenous VRCZ were continued for a total of 4 weeks and 3 weeks, respectively, and the patient was discharged on day 32 after transfer with continuing oral treatment with VRCZ. VRCZ was discontinued 5 months after the initiation of treatment because of poor medication compliance and the side effect of alopecia, and subsequent recurrence of the infection has not been confirmed.

## Discussion and conclusions

Congenital bronchial atresia is a rare pulmonary abnormality that was first described by Ramsey et al. in 1953, which is characterized by an atretic bronchus that does not communicate with the central airways [1]. It has been hypothesized that congenital bronchial atresia occurs as a result of focal ischemia of the developing bronchus due to vascular injury at 15 weeks gestation [3]. Secondary bronchial atresia has been reported to be caused by hypertrophy of bronchial mucosa, external compression of bronchi by abnormal vascular flow, and tuberculosis [4, 5]. Although the precise prevalence of congenital bronchial atresia is unclear, it is estimated to occur in 1.2 per 100,000 males [6]. It is more common in male than in female patients (male:female = 16:9) [7]. The condition often occurs at either the segmental or the subsegmental levels, and rarely occurs at the lobar level [8]. Based on CT findings, bronchial atresia was presumed to be at the subsegmental level in this case.

In patients with congenital bronchial atresia, the lung distal to the atretic bronchus becomes hyperinflated due to collateral ventilation through pores of Kohn and canals of Lambert, which results in emphysematous changes. A mucocele can also be found distal to the atretic point. Reflecting these changes, CT imaging typically reveals a mucocele, ectatic bronchi, and emphysematous changes [9]. The identification of the blind-ending bronchus via bronchoscopy is also helpful for diagnosis. However, Wang et al. reported that only 50% of patients with congenital bronchial atresia could be diagnosed using bronchoscopy [2]. In this case, we could not visually confirm the atretic bronchus on bronchoscopy.

In the present case, the presence of any disease capable of causing secondary bronchial atresia was not confirmed; therefore, the patient was likely affected by the congenital form of the disorder. Additionally, mucoceles were considered to have developed to the abscess. Ectatic bronchi and emphysematous changes occurred in the left lower lobe of the lung. These findings were confirmed by histology; however, they were not confirmed by CT presumably because of lung compression caused by the abscess.

Congenital bronchial atresia has been associated with congenital, non-pulmonary anomalies, such as pectus excavatum [10]. The disorder is typically asymptomatic [11] because infections are less likely to develop throughout the involved areas as a result of disrupted communication with the central bronchus. The most commonly reported clinical manifestation in symptomatic patients is recurrent pulmonary infection [2]. Although most infections are bacterial, *Mycobacterium avium* and cytomegalovirus infections have been reported to be infectious complications in patients with congenital bronchial atresia [12, 13]. Additionally, pneumothorax can develop due to rupture of hyperinflated lung tissue [14], and pulmonary hypertension has been reported to result from the ventilation/perfusion mismatch within hyperinflated lobes of patients [15]. To our knowledge, there has been only one report of a case of chronic pulmonary aspergillosis in patients with congenital bronchial atresia [16]. Therefore, fungal infections in bronchial atresia are presumed to be rare. In this case, the patient had an underlying disease of breast cancer, which could cause immunodeficiency. Furthermore, the patient was asymptomatic, and the disease was coincidentally observed. In contrast, in our case, the patient was immunocompetent with no underlying immunocompromising diseases and had a symptom of persistent fever. Considering that cell-mediated immune defects are a risk factor for fungal lung abscesses [17], abscess formation as a result of the fungal infection in this patient with no immune dysfunctions was unique.

No standardized guidelines for the treatment for bronchial atresia have been created. Some clinicians prefer to perform surgery on asymptomatic patients to prevent long-term consequences of the disorder, such as compression and damage to intact adjacent lung parenchyma [18]. However, follow-up with regular chest X-rays is a reasonable strategy for monitoring asymptomatic patients. Surgical resection should be considered in cases with recurrent and severe symptoms of infection, that cannot be treated effectively pharmacologically, and to exclude the possibility of the formation of malignant lesions [2]. The best duration of treatment suited for eliminating an *Aspergillus* lung abscess also remains undetermined. A minimum of 6 months of antifungal therapy is recommended for chronic pulmonary aspergillosis. In addition, surgical resection is also recommended for localized disease [19]. We treated the patient using a combination of antifungal and surgical treatment similar to recommendations for the treatment of chronic pulmonary aspergillosis. Subsequently, the disease followed a benign course despite the early discontinuation of antifungal treatment.

In conclusion, we experienced a case of congenital bronchial atresia complicated by a lung abscess due to an *A. fumigatus* infection. Supposedly, this case was unique compared with the previously reported case in that the fungal infection occurred in an immunocompetent patient and was symptomatic. Congenital bronchial atresia is a rare disease and is usually asymptomatic. However, it can cause pulmonary infections including fungal infections. Therefore, when infections occur in patients with bronchial atresia, physicians should consider fungal infections especially when antimicrobial treatment is ineffective.

## Data Availability

The data that support the findings of this case are available from the corresponding author upon reasonable request.

## Abbreviations

CT: Computed tomography
MEPM: Meropenem
VRCZ: Voriconazole
